# Exploring intraoperative hemorrhage and early recurrence in pelvic malignant solitary fibrotic tumors: a case report and literature review

**DOI:** 10.3389/fonc.2025.1531597

**Published:** 2025-05-27

**Authors:** Dong-Mei Li, Dan Tang, Ming-Rong Qie, Min-Min Hou

**Affiliations:** ^1^ Department of Obstetrics and Gynecology, West China Second University Hospital, Sichuan University, Chengdu, China; ^2^ Key Laboratory of Birth Defects and Related Diseases of Women and Children, Sichuan University, Ministry of Education, Chengdu, China

**Keywords:** malignant solitary fibrous tumor, recurrence, treatment, lymph node metastasis, case report

## Abstract

**Introduction:**

Solitary Fibrous Tumor (SFT) is a rare mesenchymal tumor characterized by CD34-positive dendritic stromal cells that can differentiate into fibroblasts or myofibroblasts. Although commonly found in the pleura, these tumors can also occur in other locations, including the retroperitoneum, where a subset may show malignant behavior, leading to local invasion and metastasis.

**Case presentation:**

We report the case of a 60-year-old woman with progressive abdominal distension (3 months) and dysuria (2 months). Imaging revealed a 7.6cm × 10.1cm × 10.7cm right pelvic mass compressing the ureter and bladder. Surgical resection of the firm, hypervascular uterine mass with infiltrative margins was achieved. Immunohistochemistry demonstrated diffuse positivity for CD34, STAT6, Bcl-2, CD99, SW-1 and ER, with focal TP53 expression and 10% Ki-67 index, confirming malignant solitary fibrous tumor. Early postoperative surveillance MRI at one month detected local recurrence with regional lymph node metastases. Given the tumor’s aggressive biology, high operative risk for re-intervention, and lack of effective systemic therapy options, a multidisciplinary team recommended transition to palliative care. The patient remains alive after following up 8 months.

**Conclusion:**

Neoadjuvant radiotherapy may benefit high-risk SFT cases nearing the limits of resectability, but current evidence highlights the challenges in managing aggressive pelvic malignant SFTs and emphasizes the need for ongoing research into effective treatment options while also aiming to stimulate discussion among scholars and encourage the sharing of experiences from similar cases to provide valuable insights for future diagnosis and treatment of malignant SFTs.

## Introduction

Solitary Fibrous Tumor (SFT) is a rare type of mesenchymal tumor initially identified in the pleura, but it can also develop in various other sites, including the pelvis, retroperitoneum, and meninges (1). These tumors are primarily composed of CD34-positive stromal cells, which can differentiate into fibroblasts or myofibroblasts (2). While SFTs generally grow slowly, malignant variants pose significant clinical challenges. These aggressive tumors are characterized by local infiltration, distant metastasis, and a high rate of recurrence, accounting for about 15-20% of all SFT cases (3). Notably, occurrences in extrathoracic locations, particularly in the pelvis, are infrequent, with only 11 documented cases of pelvic malignant SFTs to date. This paper presents a case of a pelvic malignant SFT that exhibited suspected recurrence and lymphatic metastasis one month after surgery. Furthermore, it reviews existing literature to discuss treatment strategies for managing such patients, aiming to enhance clinical understanding and management of this challenging condition.

## Case presentation

**Figure 1:**
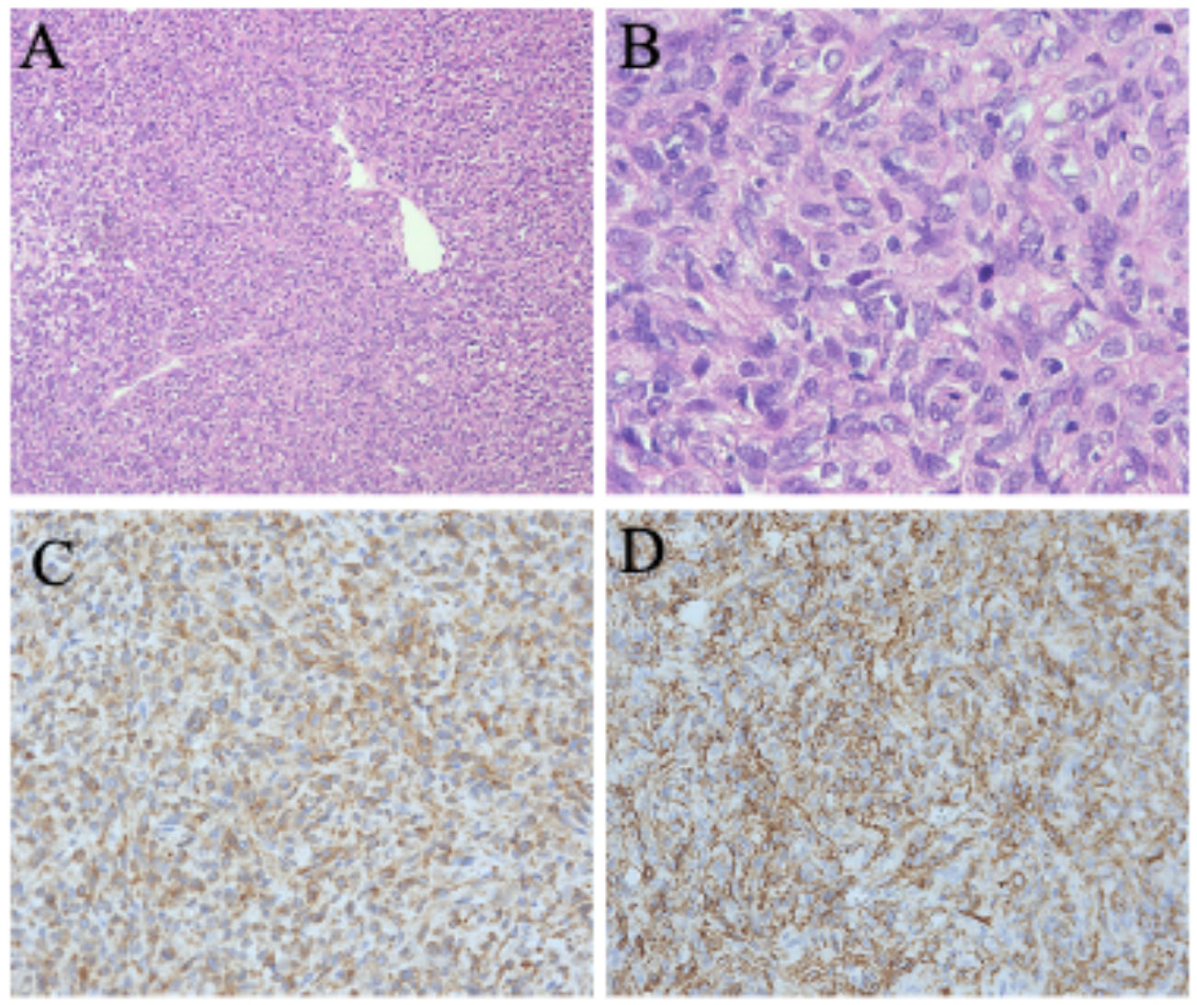
**(A)** Hematoxylin and eosin stain (100×): the tumor contains a considerable amount of collagen fibers with an increased cell density. **(B)** Hematoxylin and eosin stain (400×): there is evidence of cellular atypia, nuclear mitotic figures, and focal tumor necrosis Immunohistochemical stains (200×) are positive for CD34 **(C)** and Bcl-2 **(D)**.

**Figure 2:**
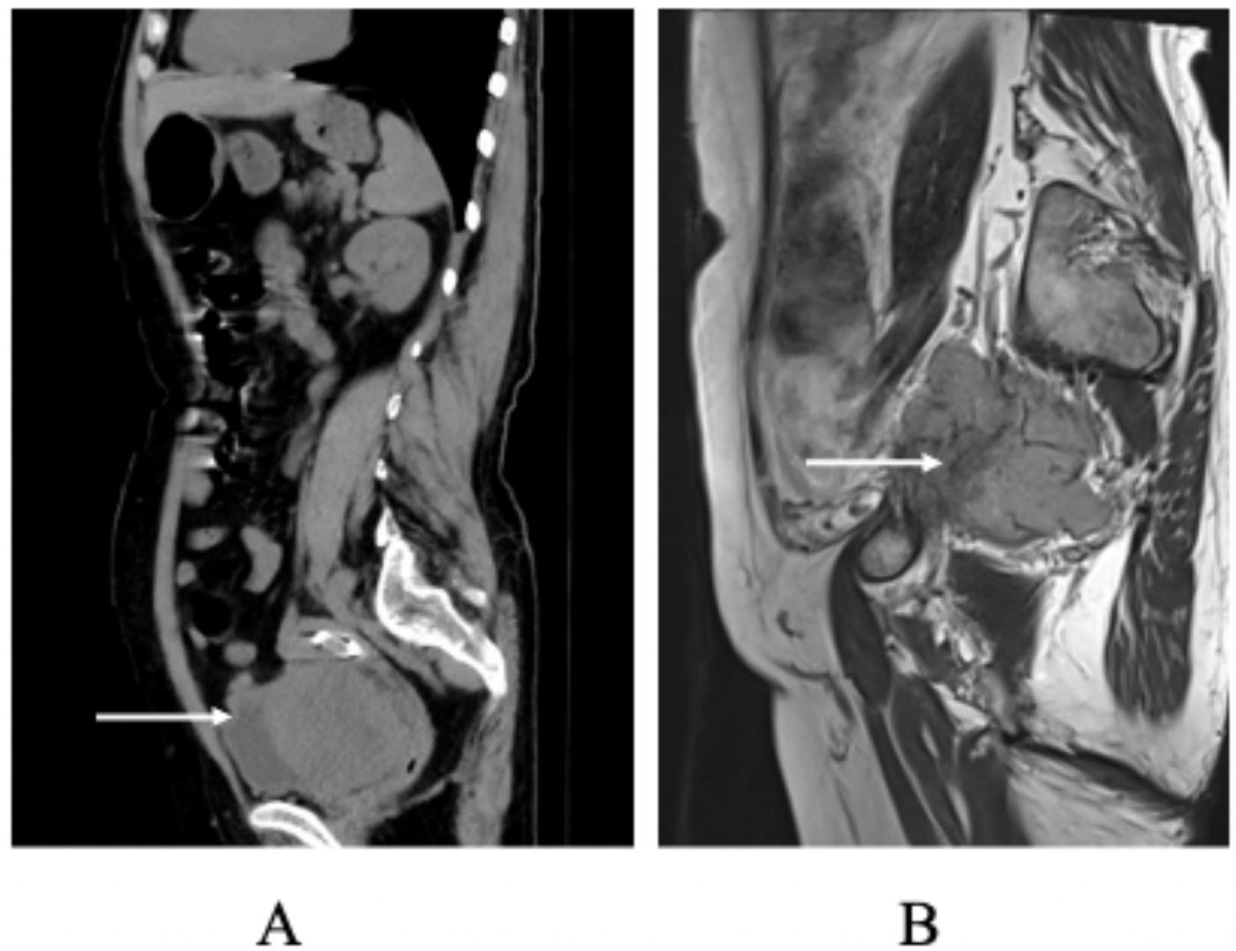
**(A)** Preoperative Non-contrast Abdominal CT Scan: this image depicts the non-contrast abdominal CT scan of the patient obtained before surgical intervention. **(B)** Postoperative MRI Scan at One Month: MRI was performed one month postoperatively to consider tumor recurrence.

This case involves a 60-year-old woman who presented with a three-month history of abdominal distension and a two-month history of dysuria. An ultrasound examination indicated a mass in the adnexal region. Screening for cervical cancer and tumor marker tests revealed no significant abnormalities. A CT scan of the pelvis and abdomen revealed a right-sided mass measuring approximately 7.6cm × 10.1cm × 10.7cm, with indistinct tumor borders between the lesion, the uterine wall, and the posterior bladder. Additionally, the lower segment of the right ureter was infiltrated, leading to hydronephrosis and dilation of the right ureter and kidney. The right adnexa appeared ambiguous, raising suspicion of a malignant ovarian tumor. During surgery, a solid mass measuring approximately 15cm × 15cm × 10cm was palpated in the lower anterior wall of the uterus and anterior to the vagina. The mass was hard, with prominent blood vessels and a rich blood supply, displacing the bladder and extending toward the right pelvic wall, pelvic floor, and vagina, with indistinct boundaries with the bladder, right ureter, and right pelvic wall. Intraoperative examination confirmed complete macroscopic resection; Histopathology confirmed complete tumor excision with negative margins, including the pelvic sidewall (R0 resection). Intraoperative blood loss was significant, totaling around 3000 ml. However, no abnormalities were detected in routine coagulation tests before surgery, our conclusion that the hemorrhage was primarily associated with the tumor’s biological behavior. The tumor showed aggressive features with vascularity and invasion into adjacent structures. Radical resection would have required bladder resection and ureteral reimplantation. After consulting the family about the risks and quality-of-life impacts, they declined procedures that could affect genitourinary function. Pathological examination confirmed a malignant SFT. (**Figure 1**) Immunohistochemical analysis showed positive results for CD34, STAT6, Bcl-2, CD99, SW-1, and ER, with occasional positivity for TP53, and Ki67 (10%). Other markers were negative, including HMB45, S-100, SMA, Caldesmon, Malan, Desmin, CD177, and DOG. One-month post-surgery, a follow-up MRI revealed an abnormal mass on the right side of the pelvis measuring approximately 6.9cm × 7.1cm × 6.0cm, accompanied by enlarged right obturator lymph nodes (about 2.4cm × 2.0cm), indicating a high likelihood of tumor recurrence and lymph node metastasis (**Figure 2**).

Given the patient’s history, we chose not to proceed with a second surgery for three primary reasons. First, the significant bleeding experienced during the initial operation heightened the risk of hemorrhage in any subsequent procedure. Second, the patient developed lymphatic metastases rapidly, indicating a progression that made reoperation inadvisable in the near term. Lastly, we organized a multidisciplinary team (MDT) discussion involving the gynecology, oncology, pathology, and imaging departments, and it was not recommended to undergo surgery again. After the discussion, we concluded that the malignancy of this disease is extremely high and progresses rapidly, and our hospital currently has no suitable radiotherapy or chemotherapy options. We recommended that the patient seek possible clinical trials at the specialized cancer centers. Subsequently, we followed up with the patient by phone. However, due to the lack of suitable radiotherapy or chemotherapy options in the specialized cancer centers, the patient received two cycles of an investigational chemotherapy regimen combining azithromycin and dacarbazine, which showed limited clinical response. Due to the lack of therapeutic efficacy, the chemotherapy regimen was discontinued, and the patient was subsequently transitioned to palliative care. Over the 8-month follow-up period, the patient remains alive.

## Discussion

This study presents a malignant SFT case with distinctive clinical characteristics. Through a comprehensive review of malignant SFTs reported across various anatomical locations, (**Table 1**) we identified that our case demonstrates an exceptionally aggressive clinical course, characterized by unexpected rapid recurrence within just one month post-operation. This accelerated disease progression challenges current clinical surveillance protocols for high-risk SFTs. Studies have identified several factors as reliable indicators of poor prognosis in malignant SFTs (11, 15). These include nuclear atypia, a high mitotic count (greater than 4 per 10 high-power fields), tumor size exceeding 10 cm, and the presence of necrosis or hemorrhage. Additionally, specific immunohistochemical markers such as CD34, CD99, Bcl-2, and STAT-6 have demonstrated high sensitivity and specificity for SFTs, while TP53 positivity is linked to malignant cases (16, 17). In the present case, the intraoperative specimen appeared as a spherical mass measuring 15cm × 15cm × 10cm. Histopathological examination revealed an increased mitotic count (7/10 HPFs), marked nuclear atypia, and focal necrosis. Immunohistochemical analysis demonstrated diffuse positivity for CD34, CD99, Bcl-2, and STAT-6, with focal TP53 expression. These findings are consistent with previously reported features of malignant solitary fibrous tumors. Currently, there is no conclusive literature addressing how the presence or absence of specific immunohistochemical markers may influence future clinical decision-making. Investigating this potential correlation represents a promising direction for further research.

**Table 1:** Clinicopathological features of malignant solitary fibrous tumors in rare sites.

Author (year)	Years	Gender	Tumor location and size (in cm)	Pathologic features	Treatment	Follow-up and outcomes
Muñoz, Eva(2008) (4)	35	Male	Left sacrum: 5	Positive for CD34 vimentin as well as for bcl-2; S-100 negative for EMA antigen; Ki-67 (∼1%)	Palliative radiotherapy and systemic chemotherapy	Follow-up shows stable disease persisting for more than one year
Yujie Cui(2010) (5)	67	Male	Right kidney: 20 Left kidney: 6	Positive for vimentin, EMA, SMA, CD34, P16, CD99,STAT6, and Bcl-2; negative for CKpan, SSTR2, S100, HMB45, desmin, PAX-8, MDM2, PR, GFAP, actin ;Ki-67 (about 10%+),	Gave up surgical treatment, underwent drug treatment	Survived for 30 months, died of a lung infection
Chang Kwon Park(2011) (6)	77	Female	Pleural tumors: 4.5	Positive for CD34, CD99, Bcl-2, vimentin, negative for Cytokeratin, EMA, SMA, S-100.	Only surgical excision	Follow up 24 year, underwent six surgical resections for six recurrent SFTs, the last recurrence is malignant
Hanping Wang(2011) (7)	6	Boy	Appendical mesentery: 10	Positive for CD34; negative for OG-1, Bcl-2, CD117, desmin, S-100 protein, and SMA.	Only surgery	Relapsed 5 months after the postoperative; died of the recurrent tumor with liver dissemination 7 months after the reoperation
Jordan Dozier(2015) (8)	41	Male	Bladder serosa: 21	Positive for vimentin, CD34 , BCL-2 and beta-catenin; negative for pan Cytokeratin, p63, Calretinin, SMA, desmin, S100, CD-31, CD-117, DOG1, EMA, STAT6, GRIA2 and WT-1;	Only surgery	Follow up eight months with no recurrence.
Brigida Iorio(2019) (9)	42	Female	Sublingual gland: 3	Positive for CD34, CD99 and ALDH1 and bcl2; negative for STAT6, S100	Only surgery	Unclear
Kumar, Pravin(2023) (10)	71	Male	Maxilla: 5	Positive for CD34, Bcl2, CD99, vimentin; negative for desmin, S-100, p63, CK5/6.	Palliative radiation therapy	Unclear
Yonghui Wang(2023) (11)	48	Female	Right breast: 25	Positive for CD34, Bcl-2, β-catenin and STAT6; negative for desmin, S-100, p63, and smooth muscle actin; Ki67 (10%)	Only surgical excision	Follow up 1 year, no recurrence
Fatema Bunajem(2023) (12)	64	Male	Right inguinal region: 9	Positive for CD34, CD99; negative for S100, HMB45, BCL2, CD31, AE/AE3, CK7, 34BetaE12, SMA, MSA, MyoD1, p63	Only surgery	Free of recurrence for two years postoperatively
Xiao-Jie Wang(2024) (13)	60	Male	The joint cavity of the left knee: uncler	Positive for CD34, Bcl-2, STAT6, EMA, beta-catenin, SMA, CK(AE1/AE3), CAM5.2; negative for desmin, S-100; Ki-67 (30% +)	Only surgery	Follow up 11 months, and the patient had multiple bone metastases
Atl Simon Arias Rivera(2024) (14)	43	Male	Pancreatic head: 12	Positive for STAT-6 and CD34; negative for CD117, DOG-1, s-100 protein, SOX-10, and CKAE1/AE3	Only surgery	Follow up three months with no recurrence.

The primary treatment for SFTs is complete surgical resection, as incomplete removal significantly increases the risk of recurrence (18, 19). In some instances, patients have undergone multiple surgical resections over extended periods, with reports of as many as six procedures spanning 24 years (6). The tumor showed aggressive features including marked vascularity, invasion into adjacent structures (bladder/ureter/pelvic wall), and loss of tissue planes. Despite suspected organ infiltration, resection was limited due to critical location and significant bleeding (3000 mL), following family’s refusal of radical surgery. However, in the case presented here, the tumor exhibited rapid regrowth within just one month following surgery, a phenomenon not commonly documented in the English literature. The tumor demonstrated aggressive characteristics with radiologic and intraoperative evidence suggesting invasion into adjacent structures (bladder, ureter, and pelvic wall). While histopathological examination confirmed R0 resection margins, the anatomical constraints imposed by the tumor’s proximity to these vital structures - combined with the family’s decision against more radical resection - may have permitted residual microscopic disease despite negative margins, potentially contributing to the observed recurrence pattern. Pelvic SFTs are often larger than their thoracic counterparts at diagnosis, increasing the likelihood of complications due to their vascularity and proximity to critical structures such as the bladder, ureter, and pelvic vasculature.

During follow-up at another institution, the patient underwent two cycles of an investigational chemotherapy regimen comprising azithromycin and dacarbazine, which demonstrated limited efficacy. Consequently, chemotherapy was discontinued, and the patient transitioned to palliative care. Regarding soft tissue tumors, evidence for chemotherapy mainly supports anthracycline-based regimens, which have shown a progression-free survival (PFS) of 3 to 5 months (20, 21). The combination of doxorubicin and ifosfamide may extend PFS further (21). Trabectedin has also demonstrated effectiveness against metastatic soft tissue sarcomas, with PFS varying between 2.3 to 11.6 months (22, 23). Additionally, the combination of temozolomide and bevacizumab has shown anti-angiogenic properties in SFTs (24). Tyrosine kinase inhibitors offer hope for patients with unresectable or recurrent disease (25). While tyrosine kinase inhibitors hold promise for patients with unresectable or recurrent disease, their routine clinical application requires further validation. For SFT cases near the limits of resectability, particularly those with a high mitotic rate, neoadjuvant radiotherapy may offer potential benefits (26). As summarized in **Table 1**, the primary treatment for SFT remains surgical resection. However, this patient was not a suitable candidate for repeat surgery. Currently, there is no standardized chemotherapy regimen for malignant SFT. While existing literature suggests that azithromycin-based combinations—commonly used for other soft tissue sarcomas—may represent a potential therapeutic option, the impact of tumor location on treatment efficacy remains unexplored. Establishing effective adjuvant or maintenance therapies post-surgery remains a critical challenge in managing this disease.

In summary, malignant SFTs are rare. Their unpredictable recurrence rates, local invasiveness, and potential for metastasis present significant challenges in treatment. These factors underscore the complexity of managing these tumors in clinical practice. As such, long-term follow-up and monitoring for patients with malignant SFT are crucial for the early detection of recurrences and the timely implementation of appropriate interventions. The aggressive nature of pelvic malignant SFTs necessitates careful consideration in terms of management and prognosis. Current treatment guidelines do not provide comprehensive strategies tailored to these tumors, highlighting a gap in the clinical framework. Future research should aim to optimize treatment approaches to enhance patient outcomes and address the unique challenges posed by malignant SFTs. In our current research and practice, we recognize that the complexity of this case presents many unresolved mysteries in the treatment process. Therefore, through this case report, we aim to engage more scholars in discussion and invite them to share their experiences and insights from similar cases, providing valuable references for our future diagnosis and treatment.

## Data Availability

The original contributions presented in the study are included in the article. Further inquiries can be directed to the corresponding author/s.
